# The Role of Parenting Practices on the Parent Perceived Impact of Child Oral Health on Family Wellbeing

**DOI:** 10.3390/ijerph19031680

**Published:** 2022-02-01

**Authors:** Nesa Aurlene, Jyothi Tadakamadla, Amit Arora, Jing Sun, Santosh Kumar Tadakamadla

**Affiliations:** 1 Madha Dental College and Hospital, The Tamil Nadu Dr.M.G.R. Medical University, Chennai 600069, India; aurlenej@gmail.com; 2 School of Medicine and Dentistry, Griffith University, Gold Coast, QLD 4222, Australia; j.tadakamadla@griffith.edu.au (J.T.); j.sun@griffith.edu.au (J.S.); 3 School of Health Sciences, Western Sydney University, Locked Bag 1797, Penrith, NSW 2751, Australia; A.Arora@westernsydney.edu.au; 4 Translational Health Research Institute, Western Sydney University, Locked Bag 1797, Penrith, NSW 2751, Australia; 5 Discipline of Child and Adolescent Health, Sydney Medical School, Faculty of Medicine and Health, The University of Sydney, Parramatta, NSW 2145, Australia; 6 Oral Health Services, Sydney Local Health District and Sydney Dental Hospital, NSW Health, Sydney, NSW 2010, Australia; 7 Health Equity Laboratory, Adelaide, NSW 2560, Australia; 8 Menzies Health Institute Queensland, Griffith University, Gold Coast, QLD 4222, Australia

**Keywords:** parenting styles, child oral health, family impact, family wellbeing, survey

## Abstract

Background: Family characteristics and parenting practices could significantly influence child oral health and the impact of child oral health on family wellbeing. Aim: To determine the association between parenting practices and parent-perceived impact of children’s oral health condition on family wellbeing. Design: A cross-sectional survey was conducted among 1539 school children in India. Parents answered the short form of FIS (Family Impact Scale), and PCRQ (parent–child relationship questionnaire) to assess the impact of the child’s oral health on family and parenting practices, respectively. Two factors emerged on factor analysis of PCRQ: ‘Positive parenting’ and ‘Power assertive parenting’. The intra-oral examination was conducted for children to assess their dental caries experience and gingival health status. Unadjusted linear regression and hierarchical multiple regression analysis were used to determine the influence of parenting practices on FIS. Results: An increase in power assertion (B = 1.16) parenting was associated with increased FIS scores indicating a higher adverse impact of the child’s oral health on family wellbeing when parents used more power assertive parenting practices. On the other hand, an increase in positive parenting (B = −1.27) was associated with decreased FIS scores, indicating a lesser impact of child’s oral health on family wellbeing when parents used more positive parenting practices. Conclusions: Parenting practices were associated with parents’ perceptions of the effect of children’s oral health on family wellbeing.

## 1. Introduction

Studies demonstrate that oral diseases negatively influence parental emotions and may result in family conflicts [1,2]. A child’s oral condition is a source of family distress, affecting parental and family activities. The most notable impacts of a child’s poor oral health on the family include parental guilt and sadness, missing work due to having to care for the child, interruption of routine family activities, disturbed sleep, and financial difficulties in paying for the child’s dental treatment [1]. These family impacts could lead to family stress and poor family functioning, which has been established to be associated with unsatisfactory oral hygiene behaviours and poor oral health in children [3,4]. Family wellbeing and child oral health are closely interrelated, therefore it is essential to know how children’s oral health conditions may cause stress in their families.

Among contextual factors that could influence a child’s oral health outcomes, parenting practices are a relatively less examined factor in oral epidemiological studies. Parents directly influence a child’s behaviour, and certain parenting styles (permissive, authoritarian) are detrimental to a child’s oral health and oral hygiene behaviours. The limited research into parenting practices and their effects on child oral health states that authoritative or positive parenting practices, where parents consider their children’s feelings and maintain a positive relationship with their child while still enforcing rules, are associated with better dental hygiene behaviour in children [5,6,7]. On the other hand, permissive parents are very flexible with their children and rarely enforce rules that discourage bad behaviour; authoritarian parents, being the polar opposite, enforce rules strictly with little to no regard for their child’s opinions [8]. Children of authoritarian parents tend to exhibit higher caries burden and poorer oral health behaviour than those who practise authoritative parenting [5]. For instance, we found that the children of parents who used power assertion (authoritarian) parenting practices tend to exhibit poor oral hygiene and high caries experience [6].

Previous research has demonstrated a negative link between authoritative practices and parental stress among children with chronic childhood illness [9]. Conversely, parents with other types of parenting practices experience more stress concerning their child’s illness [10,11]. Therefore, it could be postulated that parenting practices influence parental stress associated with child’s oral health, and parents under stress become a potential source for family stressors leading to dysfunctional families and poor child illness management [12]. To our knowledge, no study has examined parenting practices as a factor that could potentially influence the parent-perceived impact of a child’s oral condition on the family. Extreme or severe perceptions of the impact of child oral condition on the family could add to the burden placed on the family due to the child’s disease condition and result in poor management by parents. This study aims to evaluate the effect of parenting practices on the parent-perceived impact of a child’s oral health condition on the family.

## 2. Materials and Methods

### 2.1. Study Design

A cross-sectional survey was conducted from May 2014 to March 2015 among sixth-grade school children in the district of Medak, Telangana, in India. School children between the ages of 11 and 14 years and their parents were recruited for the study using a multistage random sampling procedure. The sampling procedure has been discussed in detail in a previous publication [6]. In the first stage, 9 sub-districts (administrative divisions) were selected randomly from a total of 46 sub-districts in the Medak district. In the second stage, schools proportional to the total number of schools in each sub-district were selected randomly (36 schools out of a total of 455 schools). Lastly, all sixth-grade school children in the selected 36 schools were invited to participate in the study.

### 2.2. Ethical Considerations

The study was conducted in accordance with the ethical principles in the Declaration of Helsinki, revised in 2013. Ethical approval was granted by the Human Research Ethics Committees of Griffith University, Australia (DOH/12/14/HREC), and Panineeya Institute of Dental Science & Hospital, India (Ref No: 00126). Written informed consent was obtained from parents who agreed for their children to participate in the study. Only those children whose parents consented to the study were included. All parents approached agreed to participate in the study.

### 2.3. Sample Size Estimation

Using G* power software (version 3.1.9.2, Heinrich-Heine-Universität Düsseldorf, Düsseldorf, Germany), for linear regression analysis with two tested predictors (positive and power assertion parenting practices) and 13 total number of predictors, with 80% power, 5% confidence level and the default small effect size of 0.02, a sample size of 485 children was considered adequate for this study [13].

### 2.4. Data Collection

The data collection involved three steps: collecting data from the parents using a parent questionnaire, collecting data from the children using a child questionnaire, and clinical intra-oral examination of the children. On the principal investigator’s first visit to the schools a booklet containing the information sheet, consent forms, and the parent questionnaires were taken home by the children. On the subsequent visit, child questionnaires were administered, and a clinical intra-oral examination was performed for children who had obtained written informed consent from their parents. The clinical intra-oral examination for all children was conducted by a single calibrated examiner (SK) in the schools under natural daylight.

The parent questionnaire comprised questions on socioeconomic status (SES), family structure, and the number of children in the family. Detailed information on data collection and categorization of these variables can be accessed from our previous publication [6]. Either of the parents was allowed to complete the questionnaire.

A composite measure of SES, the Kuppuswamy scale designed for Indian communities consolidating income, education, and occupation, was used in this study [14]. Family structure was categorized as single-parent family (families with only one parent and child/children), nuclear family (families in which only a husband, a wife, and their children reside) or non-nuclear family (families in which extended family members of the husband or wife also reside). Parents also answered the Telugu translated version of the Family Impact Scale (FIS) short form, which consisted of eight items with a five-point Likert response scale. The item scores ranged from 0 (never) to 4 (every day or almost every day). This was used to assess parental perceptions of the impact their child’s oral health condition had on the family [15]. The FIS item scores were summed to obtain the total scores, with higher scores indicating a greater negative impact of the child’s oral health condition on the family. Telugu FIS was found to have satisfactory psychometric properties [16]. FIS has three subscales—parental emotions (PE), parent/family functioning (PF), and family conflict (FC), with PE comprising four items related to the emotional impact of children’s oral health on them. PF and FC both have two items, each capturing the effect of children’s oral health on family activities and family conflict, respectively. PE, PF, FC, and overall FIS were the outcomes of this study.

Parenting practices were assessed using the Telugu translated version of the 40-item parent-child relationship questionnaire (PCRQ) [17]. The PCRQ consists of five dimensions to evaluate different traits of the parent–child relationship: (1) warmth (affection and admiration for each other); (2) personal relationship (similarity, intimacy, nurturance, companionship, and prosocial behaviour); (3) disciplinary warmth (praising children, providing a rationale for rules and punishment, and involving children in decision making); (4) power assertion (dominance, physical and verbal punishment); and (5) possessiveness (protectiveness and possessiveness). When the translated version was subjected to factor analysis, all the dimensions “warmth, personal relationship, disciplinary warmth, and possessiveness”, which represent overall warmth, loaded onto one factor and was named “positive parenting”. All the items of the dimension “power assertion” which represents control, loaded onto another factor. Participants selected one response on a five-point Likert scale for each item. Cumulative scores for “positive” and “power assertive” parenting practices were obtained by adding the response scores of all items belonging to the corresponding dimension. A higher score on either dimension represented parents exercising that particular parenting practice more frequently [6]. Positive and power assertion parenting practices were the main exposure variables.

Children completed a questionnaire on their oral hygiene (tooth brushing frequency per day and dental visiting practices) and dietary practices (frequency of consumption of sweet foods and sweet drinks between meals in a day, and frequency of fresh fruit consumption). Children were examined for dental caries by a single examiner following the World Health Organization (WHO) diagnostic criteria for dental caries in deciduous and permanent dentition [18]. A lesion was identified as carious when presented with an unmistakable cavity, undermined enamel, or a clinically detectable soft wall or floor. Dental caries were quantified as the sum of decayed, missing, and filled teeth in deciduous and permanent dentition (dmft + DMFT). The Modified Community Periodontal Index (CPI) was used to assess the presence or absence of gingival bleeding in all teeth [18]. This was finally quantified as the percentage of teeth with gingival bleeding for each individual.

### 2.5. Statistical Analysis

SPSS (IBM Statistics, 22.0, IBM Corp, Armonk, NY, USA) was used for statistical analysis. At the bivariate level, unpaired *t*-tests and one way ANOVA were conducted to evaluate the association of socio-behavioural characteristics with the three subscales and overall scale of FIS. Unadjusted linear regression analyses were conducted to explore the effect of age, children’s dental caries experience, gingival bleeding (mean % of teeth with gingival bleeding), and parenting practices on total FIS and its subscales. Lastly, hierarchical multiple regression analyses were used to establish the association between parenting practices and FIS and its components after adjusting for children’s socio-demographic, family, behavioural characteristics, and oral health status. The dependent variables were overall FIS and the PE, PF, and FC subscales of FIS while parenting practices remained the main exposure variables. Socio-demographic characteristics (age, gender, and SES) were entered at step 1, family-related variables (family structure, number of children, and crowding) were added at step 2, while all oral hygiene behavioural and oral health status characteristics were entered at step 3. A *p*-value < 0.05 was considered significant.

## 3. Results

Of the 1800 questionnaires distributed to the parents, 1539 were returned (response rate of 85.5%). Overall FIS and its subscale scores in relation to socio-demographic, family environment, and behavioural characteristics are presented in Table 1. Parents of male children (5.21 ± 5.48) reported higher FIS scores than female children (3.92 ± 4.81). A similar trend was observed for all the subscales. Parents belonging to the lower SES had a higher mean FIS (5.60 ± 5.61) indicating that they perceived a higher burden of child’s oral health on their family than the middle (4.68± 5.24) and upper (3.24 ± 4.29) SES parents. Family structure and the number of children were significantly associated with the FIS scores. Single parents (5.92 ± 5.73) perceived a higher burden of their child’s oral health condition on their family than parents in nuclear (4.62 ± 5.25) or non-nuclear families (4.32 ± 5.01). Likewise, parents with five or more children (6.85 ± 5.92) felt their child’s oral health condition to have a higher negative impact on the family than parents with 1–2 (4.81 ± 5.12) or 3–4 (5.08 ± 5.52) children.

Age was found to have a positive relationship with the PE subscale and overall FIS score, indicating an increase in the negative impact of oral health on the family with an increase in the child’s age (Table 2). With increased positive parenting practices, the perceived impact of the child’s oral health on the family decreased (B = −1.33; *p* < 0.001). A similar trend was also observed for the association between positive parenting and FIS subscales. On the other hand, increase in power assertion parenting practices was associated with the increase in overall FIS (B = 0.99; *p* < 0.001), PE (B = 0.23; *p* < 0.001), PF (B = 0.45; *p* < 0.001), and FC (B = 0.30; *p* < 0.001). Those using more positive parenting practices felt less burdened by their child’s oral health condition than parents who practice power assertion parenting predominantly. The impact on PE, PF, and FC as a result of the child’s oral health condition was also lower in parents with higher levels of positive parenting than parents with power assertion parenting.

Results from the adjusted analysis presented in Table 3 demonstrated that the parenting practices were consistently associated with overall FIS and all its component subscales (PE, PF, and FC) in all the three models of the hierarchical regression analyses. For instance, after controlling for all other variables, the total family impact score decreased by 1.27 units with a unit increase in positive parenting score. In comparison, a unit increase in power assertion parenting score was predicted to increase the family impact score by 1.16 units.

## 4. Discussion

To the authors’ knowledge, this is the first study to examine the relationship between parenting practices and parent-perceived impact of child’s oral health condition on the family. In this study, parenting practices were significant predictors of total FIS and all the subscales of FIS (PE, PF, and FC). As we hypothesized earlier, an increase in power assertion parenting practices was associated with an increased adverse impact of children’s oral health condition on their families, while positive parenting practices had a positive effect. Parents and children function together as a unit in a family, and the effects of a child’s illness on the family invariably affect the parents and the child. This is reiterated by the American Academy of Pediatrics, which defines child health as “the social, physical and emotional functioning of the child and, when indicated, his or her family” [19]. Although there is no previous evidence on the relationship between parenting practices and the parent perceived impact of a child’s oral health on family, there is limited evidence to suggest that parenting styles of children with chronic illnesses significantly impact parental stress [9]. Authoritarian parents, characterized by high expectations and low responsiveness to a child’s needs, experienced significantly more parenting stress than those with other parenting styles [10,11]. Our study aligns with these findings. As the power assertion parenting practices increased, the FIS scores increased, indicating that parents’ felt their child’s oral health adversely impacted the functioning of their family when they predominantly practice power assertive parenting. Parental stress is known to be associated with family functioning, and children in dysfunctional families exhibit poor oral health and oral hygiene behaviours [3,4].

The influence of SES on FIS and its component subscales (PE and PF) was notable. Lower SES parents felt an adverse impact of their child’s oral health condition on their families more acutely than parents belonging to upper or middle SES. This is in accordance with studies conducted on the relationship between SES, parenting, and family functioning, which have found that low SES families are more likely to be dysfunctional, exposing the child to harsher and more punitive parenting with low levels of support and high levels of family conflict and family violence [20,21,22].

Interestingly, single-parent families were found to experience more family conflicts and a higher burden of child oral health condition on the family than other types of families in our study. This is similar to studies conducted in the past where it was found that single parenting increases the likelihood of family conflicts despite there being fewer people in the family [23,24]. This heightened level of conflict in single-parent families is postulated to be due to multiple factors, such as lack of objectivity in solving problems, lack of tolerance, empathy, economic and social resources, lack of another parent to help with discipline and control, and other stresses associated with single parenting. Single parents also felt more overall impact of their child’s oral health conditions on their families than parents in nuclear or non-nuclear families. This association is observed consistently in previous studies, with single parenting being associated with poor family functioning [25,26]. The other socio-demographic, family-level characteristics, behavioural characteristics, and clinical oral health statuses of children failed to predict FIS in our model.

The association between parenting practices and FIS persisted even after adjusting for all confounding variables in our study. An increase in positive parenting practices was associated with a lower burden of children’s oral health condition on their families. Increased positive parenting practices were also associated with less impact on PE, PF, and FC due to the child’s oral health condition. Previous research has attributed positive parenting personality traits such as extraversion, agreeableness, openness, and conscientiousness to authoritative parents [27]. Authoritative parents have high control and high warmth, which leads children to internalize or externalize problems to a lesser degree and exhibit fewer behavioural difficulties [28,29]. The findings from our study are consistent with a previous study that demonstrated authoritative parenting to mediate the effect of childhood chronic disease on family resilience [30]. Parents with positive parenting practices with a positive outlook on life, in general, may be able to handle the stresses of unforeseen or adverse events such as child’s poor oral health more efficiently than parents with other types of parenting practices.

Conversely, parents reporting higher power assertion practices experienced a higher adverse impact of their child’s oral health on their family. This effect was also observed for all the three subscales of the FIS after adjusting for all confounding variables. Power assertion or authoritarian parents are purported to have a high degree of neuroticism characterized by nervousness, anxiety, tension, and lack of emotional stability [27]. These characteristics do not allow power assertive parents to be sensitive to their children’s needs, and parents who exercise this form of parenting tend to be exhibit high control and low warmth. Parents who predominantly practice power assertion are highly critical, disapproving and rejecting their children’ needs and feelings, stimulating hostile and aggressive behaviour in children [28,31]. Evidence from previous research shows that authoritarian parenting correlates positively with all the dysfunctional patterns of family functioning and negatively with all the functional patterns of family functioning [32].

Children’s dental caries status did not turn out to be a significant predictor for FIS in our study. This finding is in contrary to a previous study conducted in Peru which reported a positive association between FIS and dental caries [33]. Other studies have also established the negative impact of severe dental caries in children on their families [1,34,35]. The non-significant association between dental caries and family impacts in our study could be explained by the relatively low number of children with severe dental caries in our study. All the other studies that found a significant association between dental caries and family impacts were conducted among children with advanced stages of dental caries or among children with higher caries experience.

The limited evidence indicates that positive parenting approaches work best to produce favourable oral hygiene behaviours in children, and lead to better oral health outcomes [5,36,37]. Previous research has also established that poor family functioning leads to poor oral health outcomes in children [38]. However, no study so far has examined the association between parenting practices and the impact of children’s health on the family in the context of childhood oral diseases. Our study bridges this gap and shows a relationship between parenting practices and the impact of a child’s oral health condition on families. This study further adds to the literature in establishing that parenting practices and family resilience to children’s health conditions are closely interrelated.

The strengths of the present study are concurrent assessment of the influence of demographic, family level, and behavioural determinants on FIS and its three subscales (PE, PF, FC). In addition, we have also included two of the most common clinical conditions in children (dental caries and gingivitis) as independent variables in predicting FIS. Furthermore, we included a large sample of parents and children and used a validated measure (PCRQ) to assess parenting practices that showed good psychometric properties [17]. The limitations of this study are that a cross-sectional study design was adopted, therefore the associations found cannot be considered causal. The data collection was anonymous; nevertheless, as it was largely self-reported data, the possibility of socially desirable reporting by parents cannot be excluded. Although the results are not generalizable to all Indian children, the findings presented in this study are from a representative sample of families of one Indian district.

## 5. Conclusions

Parents practising high levels of power assertion experienced more adverse impacts on the family owing to their child’s oral health condition than parents who predominantly practice positive parenting. This study reiterates that positive parenting is ideal for the betterment of children’s oral health and family wellbeing.

## Figures and Tables

**Table 1:** Distribution of study population according to gender, SES, family and behavioural characteristics and their association with parental emotions, parent/family activity, and family conflict components of Family Impact Scale (FIS) and total FIS.

	N	%	Parental Emotions Mean (SD)	Parent/Family Activity Mean (SD)	Family Conflict Mean (SD)	Total Family Impact Scale Score Mean (SD)
**Gender ^a^**						
Male	915	59.5	1.01 (1.71) *	3.19 (3.40) **	1.01 (1.65) **	5.21 (5.48) **
Female	624	40.5	0.81 (1.60)	2.42 (3.01)	0.67 (1.33)	3.92 (4.81)
**Socioeconomic status ^b^**						
Upper	341	22.2	0.59 (1.22) **	1.99 (2.65) **	0.65 (1.31) *	3.24 (4.29) **
Middle	651	42.3	0.91 (1.65)	2.84 (3.23)	0.92 (1.59)	4.68 (5.24)
Lower	547	35.5	1.16 (1.88)	3.47 (3.53)	0.96 (1.59)	5.60 (5.61)
**Family structure ^b^**						
Single parent	180	11.7	1.05 (1.70)	3.58 (3.38) *	1.28 (1.87) **	5.92 (5.73) *
Nuclear	904	58.7	0.95 (1.72)	2.83 (3.29)	0.84 (1.47)	4.62 (5.25)
Non-nuclear	455	29.6	0.84 (1.54)	2.70 (3.15)	0.78 (1.48)	4.32 (5.01)
**Number of children ^b^**						
1–2	1107	71.9	0.89 (1.63)	2.74 (3.17) *	0.84 (1.49)	4.81 (5.12) *
3–4	398	25.8	1.01 (1.77)	3.13 (3.45)	0.93 (1.64)	5.08 (5.52)
>5	34	2.2	1.08 (1.67)	4.44 (3.69)	1.32 (1.71)	6.85 (5.92)
**Crowding ^b^**						
≤1 person/room	445	28.9	0.94 (1.64)	3.05 (3.31)	0.82 (1.52)	4.82 (5.30)
1.1–2 persons/room	994	64.5	0.93 (1.70)	2.76 (3.23)	0.88 (1.53)	4.58 (5.25)
>2 persons/room	100	6.4	0.87 (1.44)	3.27 (3.42)	1.03 (1.62)	5.17 (5.12)
**Toothbrushing frequency ^b^**						
Rarely or sometimes in a week	7	0.4	0.71 (1.25)	5.14 (3.80)	1.71 (2.49)	7.57 (6.82)
Once	1415	91.9	0.94 (1.68)	2.89 (3.28)	0.88 (1.53)	4.73 (5.27)
Twice or more	117	7.6	0.73 (1.51)	2.54 (2.98)	0.73 (1.55)	4.01 (5.00)
**Time since last dental visit ^b^**						
Never	1269	82.4	0.93 (1.66)	2.87 (3.25)	0.85 (1.51)	4.67 (5.19)
More than a year ago	119	7.7	0.97 (1.79)	2.55 (2.99)	0.94 (1.69)	4.47 (5.44)
Within last one year	151	9.8	0.85 (1.63)	3.16 (3.60)	1.01 (1.60)	5.02 (5.64)
**Frequency of sweet food consumption between meals ^b^**						
Twice or more	76	4.9	0.98 (1.89)	2.84 (3.39)	0.86 (1.57)	4.69 (5.72)
Once	130	8.4	0.96 (1.69)	3.52 (3.61)	0.84 (1.50)	5.33 (5.52)
Rarely or never	1333	86.6	0.92 (1.65)	2.82 (3.22)	0.88 (1.54)	4.62 (5.20)
**Frequency of sweet drinks consumption between meals ^b^**						
Twice or more	80	5.1	1.13 (1.86)	3.46 (3.39) *	1.20 (1.95)	5.80 (5.94)
Once	327	21.2	0.95 (1.65)	3.12 (3.28)	0.82 (1.56)	4.91 (5.24)
Rarely or never	1132	73.5	0.91 (1.66)	2.76 (3.25)	0.86 (1.54)	4.54 (5.20)

* Significant at *p* <0.05, ** Significant at *p* < 0.001. ^a^ Independent samples *t*-test, ^b^ One-way ANOVA.

**Table 2:** Association of age, dental caries experience, gingival bleeding, and parenting practices with FIS, PE, PF, and FC as dependent variables.

Independent Variables	Parental Emotions B (SE)	Parent/Family Activity B (SE)	Family Conflict B (SE)	Total Family Impact Scale Score B (SE)
Age	0.08 (0.04)	0.21 (0.08) *	0.05 (0.04)	0.35 (0.14) *
Dental caries (DMFT + dmft)	−0.01 (0.02)	0.07 (0.05)	0.06 (0.02)	0.13 (0.09)
Mean % of teeth with gingivitis	−0.001 (0.002)	0.001 (0.005)	−0.002 (0.002)	−0.002 (0.008)
Positive parenting	−0.21 (0.06) *	−0.76 (0.13) **	−0.34 (0.06) **	−1.33 (0.21) **
Power assertion parenting	0.23 (0.06) **	0.45 (0.13) **	0.30 (0.06) **	0.99 (0.21) **

* Significant at *p* < 0.05, ** Significant at *p* < 0.001.

**Table 3:** Results from adjusted hierarchical regression analysis with total FIS, PE, PF & FC as dependent variables.

Independent Variables	Parental Emotions B (SE)	Parent/Family Activity B (SE)	Family Conflict B (SE)	Total Family Impact Scale Score B (SE)
Step 1 (adjusted for socio-demographic characteristics)				
Positive parenting	−0.25 (0.07) **	−0.81 (0.14) **	−0.46 (0.06) **	−1.52 (0.22) **
Power assertion parenting	0.30 (0.07) **	0.66 (0.14) **	0.43 (0.06) **	1.40 (0.22) **
Step 2 (adjusted for socio-demographic and family environment)				
Positive parenting	−0.25 (0.07) **	−0.78 (0.14) **	−0.44 (0.06) **	−1.47 (0.22) **
Power assertion parenting	0.29 (0.07) **	0.62 (0.13) **	0.43 (0.06) **	1.35 (0.22) **
Step 3 (adjusted for socio-demographic, family environment and oral health outcomes)				
Positive parenting	−0.16 (0.07) *	−0.72 (0.14) **	−0.39 (0.07) **	−1.27 (0.23) **
Power assertion parenting	0.25 (0.07) **	0.54 (0.14) **	0.37 (0.06) **	1.16 (0.23) **

* Significant at *p* < 0.05, ** significant at *p* < 0.001. Step 1—adjusted for socio-demographic characteristics (age, gender, and SES); Step 2—socio-demographic characteristics + family related variables (Family structure, number of children, and crowding); Step 3—socio-demographic + family related + oral hygiene behaviour + dental caries experience (DMFT + dmft) + gingivitis.

## Data Availability

The data presented in this study are available on request from the corresponding author.
